# Supplementary material for: Causal Relationship between Aging and Anorexia Nervosa: A White-Matter-Microstructure-Mediated Mendelian Randomization Analysis
Source: Biomedicines. 2024 Aug 16;12(8):1874. doi: 10.3390/biomedicines12081874 (PMC11351342; doi:10.3390/biomedicines12081874)
Supplement: Supplementary file 1 [file biomedicines-12-01874-s001.zip › Supplementary Figure/Figure S4/Singe SNP plot of MR analysis for TL to ubm-b-2089.pdf]

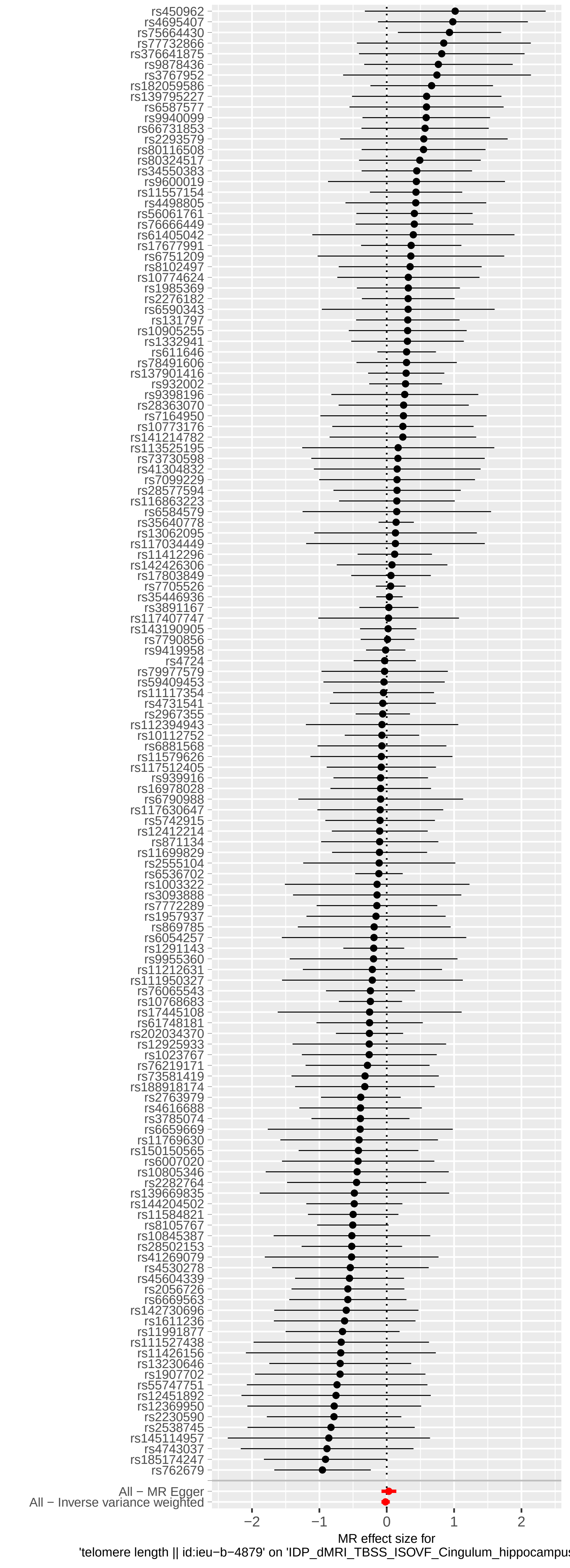
rs450962
rs4695407
rs75664430
rs77732866
rs376641875
rs9878436
rs3767952
rs182059586
rs139795227
rs6587577
rs9940099
rs66731853
rs2293579
rs80116508
rs80324517
rs34550383
rs9600019
rs11557154
rs4498805
rs56061761
rs76666449
rs61405042
rs17677991
rs6751209
rs8102497
rs10774624
rs1985369
rs2276182
rs6590343
rs131797
rs10905255
rs1332941
rs611646
rs78491606
rs137901416
rs932002
rs9398196
rs28363070
rs7164950
rs10773176
rs141214782
rs113525195
rs73730598
rs41304832
rs7099229
rs28577594
rs116863223
rs6584579
rs35640778
rs13062095
rs117034449
rs11412296
rs142426306
rs17803849
rs7705526
rs35446936
rs3891167
rs117407747
rs143190905
rs7790856
rs9419958
rs4724
rs79977579
rs59409453
rs11117354
rs4731541
rs2967355
rs112394943
rs10112752
rs6881568
rs11579626
rs117512405
rs939916
rs16978028
rs6790988
rs117630647
rs5742915
rs12412214
rs871134
rs11699829
rs2555104
rs6536702
rs1003322
rs3093888
rs7772289
rs1957937
rs869785
rs6054257
rs1291143
rs9955360
rs11212631
rs111950327
rs76065543
rs10768683
rs17445108
rs61748181
rs202034370
rs12925933
rs1023767
rs76219171
rs73581419
rs188918174
rs2763979
rs4616688
rs3785074
rs6659669
rs11769630
rs150150565
rs6007020
rs10805346
rs2282764
rs139669835
rs144204502
rs11584821
rs8105767
rs10845387
rs28502153
rs41269079
rs4530278
rs45604339
rs2056726
rs6669563
rs142730696
rs1611236
rs11991877
rs111527438
rs11426156
rs13230646
rs1907702
rs55747751
rs12451892
rs12369950
rs2230590
rs2538745
rs145114957
rs4743037
rs185174247
rs762679
All − MR Egger
All − Inverse variance weighted
−2
−1
0
1
2
MR effect size for
'telomere length || id:ieu−b−4879' on 'IDP_dMRI_TBSS_ISOVF_Cingulum_hippocampus
